# Risk factors and prediction model of sleep disturbance in patients with maintenance hemodialysis: A single center study

**DOI:** 10.3389/fneur.2022.955352

**Published:** 2022-07-26

**Authors:** Rongpeng Xu, Liying Miao, Jiayuan Ni, Yuan Ding, Yuwei Song, Chun Yang, Bin Zhu, Riyue Jiang

**Affiliations:** ^1^Department of Critical Care Medicine, The Third Affiliated Hospital of Soochow University, Changzhou, China; ^2^Department of Critical Care Medicine, Zhejiang Provincial People's Hospital, Hangzhou, China; ^3^Department of Nephrology, The Third Affiliated Hospital of Soochow University, Changzhou, China; ^4^Department of Anesthesiology and Perioperative Medicine, The First Affiliated Hospital of Nanjing Medical University, Nanjing, China; ^5^Department of Radiation Oncology, The First Affiliated Hospital of Nanjing Medical University, Nanjing, China

**Keywords:** maintenance hemodialysis, sleep disturbance, sleep quality, factors influencing, prediction model

## Abstract

**Objectives:**

This study aimed to explore the risk factors and develop a prediction model of sleep disturbance in maintenance hemodialysis (MHD) patients.

**Methods:**

In this study, 193 MHD patients were enrolled and sleep quality was assessed by Pittsburgh Sleep Quality Index. Binary logistic regression analysis was used to explore the risk factors for sleep disturbance in MHD patients, including demographic, clinical and laboratory parameters, and that a prediction model was developed on the basis of risk factors by two-way stepwise regression. The final prediction model is displayed by nomogram and verified internally by bootstrap resampling procedure.

**Results:**

The prevalence of sleep disturbance and severe sleep disturbance in MHD patients was 63.73 and 26.42%, respectively. Independent risk factors for sleep disturbance in MHD patients included higher 0.1^*^age (OR = 1.476, 95% *CI*: 1.103–1.975, *P* = 0.009), lower albumin (OR = 0.863, 95% *CI*: 0.771–0.965, *P* = 0.010), and lower 10^*^calcium levels (OR = 0.747, 95% *CI*: 0.615–0.907, *P* = 0.003). In addition, higher 0.1^*^age, lower albumin levels, and anxiety were independently associated with severe sleep disturbance in MHD patients. A risk prediction model of sleep disturbance in MHD patients showed that the concordance index after calibration is 0.736, and the calibration curve is approximately distributed along the reference line.

**Conclusions:**

Older age, lower albumin and calcium levels are higher risk factors of sleep disturbance in MHD, and the prediction model for the assessment of sleep disturbance in MHD patients has excellent discrimination and calibration.

## Introduction

Chronic kidney disease (CKD) is a global public health problem with increasing incidence and prevalence (1). According to statistical analyses, the prevalence of CKD in China is about 10.8%, and it is as high as 18.3% in some areas of China (2). When CKD progresses to the end stage, the main treatment options for patients are maintenance hemodialysis (MHD) and kidney transplantation. At the same time, MHD has become the main treatment for patients with end-stage kidney disease (ESKD) due to the serious shortage of donors for kidney transplantation. However, accumulating evidence indicates that sleep disturbance is frequently observed in MHD patients, and the prevalence rate is up to 49–98% (3–5). In addition, studies have found that sleep disturbance can lead to poor quality of life in MHD patients (6), as well as an increased risk of cardiovascular disease and all-cause mortality (7, 8). Unfortunately, sleep disturbance in MHD patients have not attracted the attention of clinicians, and lack of effective diagnosis and treatment (9). Therefore, more studies are needed to focus on sleep disturbance in MHD patients, in order to find better management strategies for the clinic.

Sleep occupies approximately one-third of human life span and is crucial for body health (10). However, the occurrence of sleep disturbance is common. Studies have found that sleep disturbance is a risk factor for of CKD (11), and is also related to the progression of CKD to ESKD (12). In MHD patients, there are various manifestations of sleep disturbance, including insomnia, restless legs syndrome (RLS) and sleep-related breathing disorders (4). As mentioned above, sleep disturbance has adverse effects on MHD patients. However, the mechanism of sleep disturbance in MHD patients is still unclear, and many phenomena are difficult to be explained by sleep itself. Therefore, it is important to explore the factors to improve sleep quality.

Currently, a series of studies have collected the demographic parameters and laboratory indexes of MHD patients to analyze the factors of sleep disturbance, and obtained different results. A study has found that gender, age, education level, diabetes history, blood phosphorus level and depression are associated with the sleep quality of MHD patients (13). Another study has confirmed that compared with MHD patients with good sleep, there are more males, lower serum parathyroid hormone (PTH) and 25-hydroxy vitamin D levels, and a higher incidence of depression in patients with poor sleep, while there is no significant difference in age between groups (14). Although both studies showed that gender and depression were associated with sleep disturbance in MHD patients, there were differences in age. In addition, a study has showed that old age and low serum selenium level are risk factors for sleep disturbance in MHD patients, and also found that high serum PTH level is associated with sleep disturbance in MHD patients (15). This contradictory result has blurred the role of serum PTH level in the sleep disturbance associated with MHD. Furthermore, studies have shown that diet regulation and melatonin can improve the sleep quality of MHD patients (16, 17). These lines of evidence suggest that there are many factors associated with sleep disturbance in MHD patients, and the role of some factors, such as age and PTH, needs to be confirmed by more studies.

In addition, few studies on factors influencing severity of sleep disturbance in MHD patients have been reported. However, a previous study has found that the severity of sleep disturbance in hemodialysis patients is positively correlated with the severity of depression and negatively correlated with the quality of life (18), which indicates that the severity of sleep disturbance can directly affect the prognosis of MHD patients. Moreover, no study has established a prediction model for the risk of sleep disturbance in MHD patients, which leads to the failure of early intervention. Therefore, exploring the influencing factors of the severity of sleep disturbance and developing a prediction model for the risk of sleep disturbance may be a key strategy to improve the sleep quality of MHD patients.

The purpose of this study was to explore the risk factors and to construct a risk prediction model of sleep disturbance in MHD patients, in order to provide strategies for the prevention and treatment of sleep disturbance in MHD patients.

## Methods

### Study participants

This was a single-center and cross-sectional study involving 193 MHD patients who underwent hemodialysis from April 2020 to March 2021 at the Blood Purification Center of the Department of Nephrology, The Third Affiliated Hospital of Soochow University. This study was approved by the Ethics Committee of the Third Affiliated Hospital of Soochow University and registered in the Chinese Clinical Trial Register (clinical trial number: ChiCTR2100042093).

The inclusion criteria were (1) age ≥ 18 years; (2) regular hemodialysis therapy >3 months; (3) ability to complete questionnaires on sleep disturbance, anxiety and depression; (4) signed an informed consent form. The exclusion criteria were (1) the history of sleep disturbance before CKD; (2) the history of dementia, anxiety, depression, Alzheimer's disease or schizophrenia before CKD; (3) A history of trauma, surgery or infection within the past 3 months; (4) complicated with malignant tumor; (5) declined to participate.

All MHD patients enrolled in the study received blood purification 3 times per week (hemodialysis once weekly plus hemodiafiltration twice weekly or hemodialysis twice weekly plus hemodiafiltration once weekly). The low-flux polysulfone membrane dialyzer (B. Braun Diacap LOPS15, Germany) was used for hemodialysis and the high-throughput polysulfone membrane dialyzer (B. Braun Diacap HIPS15, Germany) was used for hemodiafiltration. Each blood purification treatment was ~4 h and low molecular weight heparin was used for anticoagulation. The dialysate was bicarbonate with a flow rate of 500 ml/min, and the average blood flow velocity was 200–280 ml/min. Displacement volume using post-replacement was calculated by ~30% of the ultrafiltration flow rate.

### Data collection

Patients were informed of the study's objectives and instructed to complete questionnaires. The data collection instruments were structured questionnaires, the Hospital Anxiety and Depression Scale (HADS), and the Pittsburgh Sleep Quality Index (PSQI).

### Demographic, clinical, and laboratory data

Demographic and clinical data were collected from face-to-face interviews conducted by trained nurses using structured questionnaires when MHD patients were awaiting hemodialysis treatment. The following information was recorded: age, duration of dialysis, gender, body mass index (BMI), primary diseases, smoking and alcohol consumption, marital status, educational level, history of hypertension, history of diabetes mellitus, and history of cardiovascular and cerebrovascular diseases. Venous blood samples were collected before the hemodialysis. The following laboratory parameters were measured: hemoglobin (Hb), white blood cell (WBC), red blood cell (RBC), hematocrit (Hct), platelet (PLT), C-reactive protein (CRP), triglyceride (TG), total cholesterol (TC), high-density lipoprotein cholesterol (HDL-C), low-density lipoprotein cholesterol (LDL-C), albumin (Alb), creatinine (Cr), blood urea nitrogen (BUN), serum phosphorus (P), calcium (Ca^2+^) and PTH.

### Depression and anxiety

The HADS includes 14 items assessing depression and anxiety in general hospital patients. It was divided into depression subscale (HADS-D) and anxiety subscale (HADS-A). The two subscales each contain 7 items, with scores ranging from 0 to 3 points (19). The score for each subscale is computed by total score of the corresponding 7 items (0–21 points). The recommended cut-off is 8 points for anxiety or depression, with a higher score indicating more severe depression or anxiety. The HADS has acceptable reliability in Chinese (Cronbach's α = 0.776) (20).

### Sleep quality

The PSQI was used to assess the sleep quality of the subjects over the past month (21). The PSQI included 19-items that are composed of seven components: subjective sleep quality, sleep latency, total sleep duration, habitual sleep efficiency, sleep disturbance, use of sleep drugs and daytime dysfunction. Each component was scored 0 to 3 points, with a score greater than 1 indicating a sleep problem in that component. The total scores of PSQI ranged from 0 to 21, with greater than 5 indicating sleep disturbance in the last month, and greater than 10 indicating severe sleep disturbance in the last month (15). The PSQI has acceptable reliability in Chinese (Cronbach's α = 0.713) (22).

### Statistical analysis

In the analysis of factors influencing, SPSS 25.0 (IBM Corp., Armonk, NY, USA) was used for statistical analyses and GraphPad Prism 8 (GraphPad Software Inc., San Diego, CA, USA) was used for plotting. The normality of data distribution was assessed using the Shapiro-Wilk test. Continuous variables with normal distribution were shown as mean ± standard deviation, and data with a skewed distribution were shown as medians (25–75% interquartile ranges); categorical variables were expressed as frequency (percentage). The differences between the two groups were evaluated by the Student's *t* test, Mann–Whitney U-test or chi-squared test. According to the actual clinical significance, variables with *P* < 0.1 in the comparison between groups were transformed and analyzed by univariate binary logistic regression analysis. Variables with *P* < 0.5 in univariate binary logistic regression analysis were further included in multivariate binary logistic regression analysis to obtain independent risk factors for sleep disturbance in MHD patients. A *P* < 0.05 was considered to be statistically significant.

The prediction model was developed using R software (version 4.1.2). The initial prediction model was established by using the variables with *P* < 0.1 in univariate binary logistic regression analysis and variables considered to be meaningful in previous studies, and optimized by two-way stepwise regression. A nomogram was developed based on the results of the two-way stepwise regression. The internal validation of the prediction model was carried out by 1,000 bootstrap resampling procedure. The discrimination and calibration of the prediction model were evaluated with the concordance index (C-index) and calibration curve.

## Results

### Recruitment process and general clinical characteristics of MHD patients

Of the 236 MHD patients were enrolled, 43 were excluded from this study: 11 patients with hemodialysis therapy <3 months, 13 patients failed to complete PSQI and HADS, 6 patients with a history of sleep disturbance, 3 patients with a history of dementia, 2 patients with a history of depression, 1 patient had malignant tumor, 5 patients declined to participate, and 2 patients withdrew from the study (Supplementary Figure 1).

Table 1 shows the clinical characteristics of 193 ultimately enrolled MHD patients. All participants ranged in age from 27 to 79, with an average age of 53.09 ± 11.68 years. Male patients accounted for 65.8%, and the average BMI of all patients was 21.93 ± 3.26 kg/m^2^. The main primary disease was chronic glomerulonephritis followed by hypertensive nephropathy and diabetic nephropathy. The duration of hemodialysis was <3 years in 20.7% of patients, and more than 5 years in 68.9% of patients. Smoking and alcohol consumption accounted for 26.9 and 9.8%, respectively. Almost all the patients were married and 53.4% had high school or above. Patients with hypertension, cardiovascular and cerebrovascular, and diabetes mellitus diseases were 84.5, 17.1 and 18.1%, respectively. Anxiety occurred in 15.5% of patients and depression in 17.6% of patients.

**Table 1:** Clinical characteristics of MHD patients with or without sleep disturbance.

**Variables**	**Total (*****n*** = **193)**	**Sleep disturbance (*****n*** = **123)**	**No sleep disturbance (*****n*** = **70)**	**t/Z/**χ^2^	***P*** **value**
Age (years)	53.09 ± 11.68	54.80 ± 11.50	50.09 ± 11.46	2.744	0.007^a^
Male [*n* (%)]	127 (65.8)	77 (62.6)	50 (71.4)	1.545	0.214^c^
BMI (kg/m^2^)	21.93 ± 3.26	21.98 ± 3.13	21.86 ± 3.51	0.242	0.809^a^
Primary diseases				2.984	0.394^c^
Chronic glomerulo-nephritis [*n* (%)]	40 (20.7)	24 (19.5)	16 (22.9)		
Diabetic nephropathy [*n* (%)]	26 (13.5)	18 (14.6)	8 (11.4)		
Hypertensive nephropathy [*n* (%)]	39 (20.2)	21 (17.1)	18 (25.7)		
Others [*n* (%)]	88 (45.6)	60 (48.8)	28 (40.0)		
Duration of dialysis				−1.161	0.246^b^
<3 years [*n* (%)]	40 (20.7)	22 (17.9)	18 (25.7)		
3–5 years [*n* (%)]	20 (10.4)	13 (10.6)	7 (10.0)		
>5 years [*n* (%)]	133 (68.9)	88 (71.5)	45 (64.3)		
Smoking [*n* (%)]	52 (26.9)	33 (26.8)	19 (27.1)	0.002	0.962^c^
Drinking [*n* (%)]	19 (9.8)	9 (7.3)	10 (14.3)	2.441	0.118^c^
Married [*n* (%)]	188 (97.4)	120 (97.6)	68 (97.1)	0.031	0.860^c^
High school or above [*n* (%)]	103 (53.4)	68 (55.3)	35 (50.0)	0.501	0.479^c^
Hypertension [*n* (%)]	163 (84.5)	104 (84.6)	59 (84.3)	0.002	0.961^c^
Diabetes mellitus [*n* (%)]	35 (18.1)	25 (20.3)	10 (14.3)	1.096	0.295^c^
Cardiovascular and cerebrovascular diseases [*n* (%)]	33 (17.1)	24 (19.5)	9 (12.9)	1.394	0.238^c^
Hb (g/L)	108.73 ± 18.47	108.75 ± 19.36	108.69 ± 16.92	0.022	0.982^a^
WBC (10^9^/L)	5.75 (4.85–6.92)	5.86 (4.68–6.88)	5.63 (5.04–7.01)	−0.176	0.861^b^
RBC (10^12^/L)	3.68 ± 0.66	3.71 ± 0.69	3.63 ± 0.62	0.762	0.447^a^
Hct (L/L)	0.33 ± 0.06	0.33 ± 0.06	0.33 ± 0.05	0.158	0.875^a^
PLT (10^9^/L)	177.86 ± 58.66	175.54 ± 62.01	181.93 ± 52.44	−0.726	0.469^a^
TG (mmol/L)	1.60 (1.17–2.50)	1.62 (1.25–2.40)	1.60 (1.11–2.84)	−0.405	0.686^b^
TC (mmol/L)	4.11 ± 0.97	4.05 ± 0.97	4.21 ± 0.96	−1.098	0.274^a^
HDL-C (mmol/L)	0.90 (0.76–1.15)	0.87 (0.74–1.11)	0.98 (0.80–1.16)	−1.540	0.124^b^
LDL-C (mmol/L)	2.30 ± 0.74	2.25 ± 0.73	2.38 ± 0.76	−1.168	0.244^a^
Alb (g/L)	38.30 (36.30–40.15)	38.00 (36.20–39.60)	39.55 (36.78–41.43)	−3.303	0.001^b^
P (mmol/L)	1.97 (1.61–2.34)	2.11 (1.62–2.43)	1.89 (1.59–2.26)	−1.576	0.115^b^
Ca^2+^ (mmol/L)	2.28 ± 0.18	2.25 ± 0.18	2.34 ± 01.7	−3.497	0.001^a^
CRP (mg/L)	2.70 (2.20–3.40)	2.80 (2.20–3.60)	2.60 (2.10–3.10)	−1.628	0.103^b^
PTH (ng/L)	370.60 (197.35–609.25)	388.60 (199.50–614.20)	341.25 (185.08–591.45)	−0.525	0.599^b^
Cr (umol/L)	896.08 ± 186.95	891.84 ± 188.19	903.52 ± 185.87	−0.416	0.678^a^
BUN (mmol/L)	22.08 (18.11–26.30)	22.61 (18.10–26.30)	22.02 (18.51–25.43)	−0.549	0.583^b^
Anxiety n (%)	30 (15.5)	25 (20.3)	5 (7.1)	5.905	0.015^c^
Depression n (%)	34 (17.6)	27 (22.0)	7 (10.0)	4.390	0.036^c^

*Alb, albumin; BMI, body mass index; BUN, blood urea nitrogen; Ca^2+^, calcium; Cr, creatinine; CRP, C-reactive protein; Hb, hemoglobin; Hct, hematocrit; HDL-C, high-density lipoprotein cholesterol; LDL-C, low-density lipoprotein cholesterol; MHD, maintenance hemodialysis; P, phosphorus; PLT, blood platelet; PTH, parathyroid hormone; RBC, red blood cell; TC, total cholesterol; TG, triglyceride; WBC, white blood cell*.

*^a^ Calculated by Student's t test*.

*^b^ Calculated by Mann–Whitney U-test*.

*^c^ Calculated by chi-squared test*.

### Sleep survey results in MHD patients

Table 2 shows the scores of PSQI in MHD patients. The average total PSQI score was 7.98 ± 4.74 points, with 123 (63.73%) patients > 5 points and 51 (26.42%) patients > 10 points. In addition, the average score of PSQI components in the sleep disturbance group was from high to low: sleep latency, daytime dysfunction, total sleep duration, subjective sleep quality, habitual sleep efficiency, sleep disturbance, and use of sleep drugs were 2.07 ± 0.91, 1.71 ± 0.93, 1.59 ± 1.01, 1.55 ± 0.75, 1.45 ± 1.18, 1.23 ± 0.49, and 1.03 ± 1.34 points, respectively.

**Table 2:** PSQI score of MHD patients.

**Dimensions**	**Total** **(*n* = 193)**	**Sleep disturbance (*n* = 123)**	**No sleep disturbance (*n* = 70)**
Subjective evaluation of sleep quality (points)	1.19 ± 0.82	1.55 ± 0.75	0.56 ± 0.50
>1 point	49 (25.39)		
Sleep latency (points)	1.58 ± 1.09	2.07 ± 0.91	0.71 ± 0.82
>1 point	110 (56.99)		
Total sleep duration (points)	1.17 ± 1.07	1.59 ± 1.01	0.44 ± 0.75
>1 point	82 (42.49)		
Habitual sleep efficiency (points)	0.98 ± 1.15	1.45 ± 1.18	0.17 ± 0.42
>1 point	56 (29.02)		
Sleep disturbance (points)	1.06 ± 0.54	1.23 ± 0.49	0.77 ± 0.49
>1 point	32 (16.58)		
Use of sleep drugs (points)	0.67 ± 1.19	1.03 ± 1.34	0.04 ± 0.36
>1 point	45 (23.32)		
Daytime dysfunction (points)	1.33 ± 0.97	1.71 ± 0.93	0.66 ± 0.63
>1 point	75 (38.86)		
Total PSQI score (points)	7.98 ± 4.74	10.62 ± 3.86	3.36 ± 1.44
>5 points	123 (63.73)		
>10 points	51 (26.42)		

*MHD, maintenance hemodialysis; PSQI, Pittsburgh Sleep Quality Index*.

### Clinical characteristics of MHD patients in the sleep disturbance and no sleep disturbance groups

Table 1 shows the clinical characteristics of MHD patients with or without sleep disturbance. The age (*P* = 0.007) of MHD patients in the sleep disturbance group was significantly higher than those in the no sleep disturbance group. Conversely, Alb and Ca^2+^ levels (*P* = 0.001) were significantly higher in the no sleep disturbance group than those in the sleep disturbance group. In addition, anxiety (*P* = 0.015) and depression (*P* = 0.036) were decreased significantly in the no sleep disturbance group compared with the sleep disturbance group. It should be noted that CRP and PTH levels were higher in the sleep disturbance group compared with the no sleep disturbance group, although the differences did not show a statistical significance.

Supplementary Table 1 shows the univariate binary logistic regression analyses between sleep disturbance of MHD patients and clinical characteristics. Age and Ca^2+^ were transformed into 0.1^*^ age and 10^*^Ca^2+^, respectively. The higher 0.1 ^*^ age (OR = 1.435, 95% *CI*: 1.100-1.873, *P* = 0.008), anxiety (OR = 3.316, 95% *CI*: 1.208–9.106, *P* = 0.020), and depression (OR = 2.531, 95% *CI*: 1.040–6.164, *P* = 0.041) were risk factors for sleep disturbance in MHD patients. Conversely, lower Alb (OR = 0.827, 95% *CI*: 0.743–0.921, *P* = 0.001) and 10^*^Ca^2+^ levels (OR = 0.731, 95% *CI*: 0.607–0.880, *P* = 0.001) were associated with an increased risk of sleep disturbance in MHD patients.

The multivariate binary logistic regression analysis between sleep disturbance of MHD patients and clinical characteristics (Supplementary Figure 2). Higher 0.1^*^age (OR = 1.476, 95% *CI*: 1.103-1.975, *P* = 0.009), lower Alb (OR = 0.863, 95% *CI*: 0.771-0.965, *P* = 0.010) and lower 10^*^Ca^2+^ levels (OR = 0.747, 95% *CI*: 0.615-0.907, *P* = 0.003) were independent risk factors for sleep disturbance in MHD patients.

### Clinical characteristics of MHD patients in the severe sleep disturbance and no severe sleep disturbance groups

Supplementary Table 2 shows the clinical characteristics of MHD patients with or without severe sleep disturbance. The age (*P* = 0.005) and TG (*P* = 0.020) of MHD patients in the severe sleep disturbance group was significantly higher than those in the no severe sleep disturbance group. In contrast, HDL-C (*P* = 0.017) and Alb levels (*P* = 0.006) were significantly higher in the no severe sleep disturbance group than those in the severe sleep disturbance group. Additionally, anxiety (*P* < 0.001) and depression (*P* = 0.010) were increased significantly in the severe sleep disturbance group compared with the no severe sleep disturbance group. It is worth noting that the duration of dialysis was longer in the severe sleep disturbance group compared with the no severe sleep disturbance group, although the differences did not show a statistical significance.

Supplementary Table 3 shows the univariate binary logistic regression analyses between severe sleep disturbance of MHD patients and clinical characteristics. Age was transformed into 0.1^*^ age, and duration of dialysis was converted into grade variable. The higher 0.1 ^*^ age (OR = 1.493, 95% *CI*: 1.121–1.989, *P* = 0.006), the longer the duration of dialysis [3–5 years (OR = 3.769, 95% *CI*: 1.015–14.003, *P* = 0.048), >5 years (OR = 2.904, 95% *CI*: 1.059–7.963, *P* = 0.038)], anxiety (OR = 4.180, 95% *CI*: 1.861–9.385, *P* = 0.001) and depression (OR = 2.697, 95% *CI*: 1.246–5.838, *P* = 0.012) were risk factors for severe sleep disturbance in MHD patients. The lower HDL-C (OR = 0.212, 95% *CI*: 0.058–0.774, *P* = 0.019) and Alb (OR = 0.865, 95% *CI*: 0.778–0.963, *P* = 0.008) were associated with an increased risk of severe sleep disturbance in MHD patients.

The multivariate binary logistic regression analysis between severe sleep disturbance of MHD patients and clinical characteristics (Supplementary Figure 3). Higher 0.1^*^age (OR = 1.476, 95% *CI*: 1.076–2.023, *P* = 0.016), lower Alb (OR = 0.877, 95% *CI*: 0.778–0.989, *P* = 0.032) and anxiety (OR = 3.442, 95% *CI*: 1.204–9.839, *P* = 0.021) were independent risk factors for severe sleep disturbance in MHD patients.

### The risk prediction model for sleep disturbance in MHD patients

**Figure 1:**
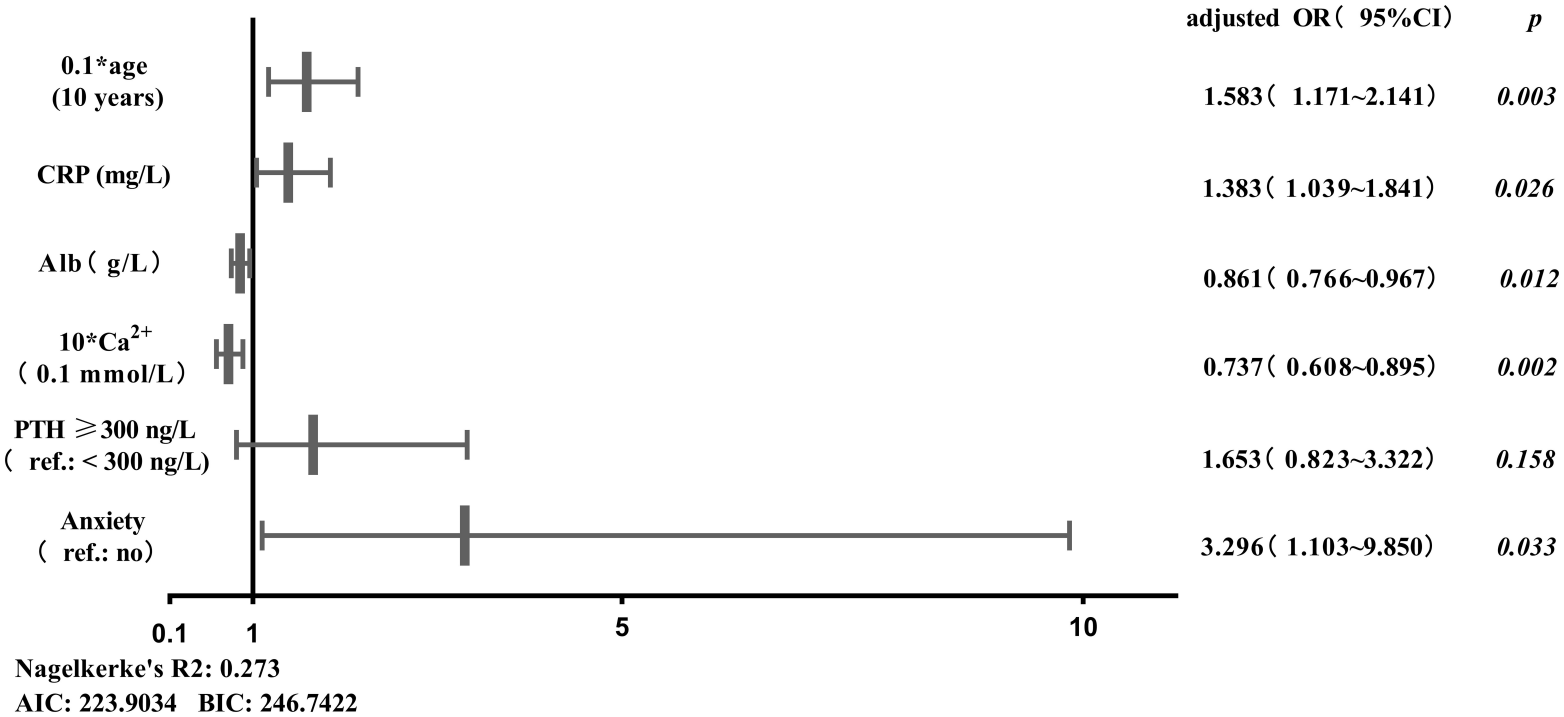
Binary logistic regression analysis between sleep disturbance of MHD patients and predictors. AIC, Akaike information criterion; Alb, albumin; BIC, Bayesian information criterion; Ca^2+^, calcium; CRP, C-reactive protein; PTH, parathyroid hormone; Ref., reference.

**Figure 2:**
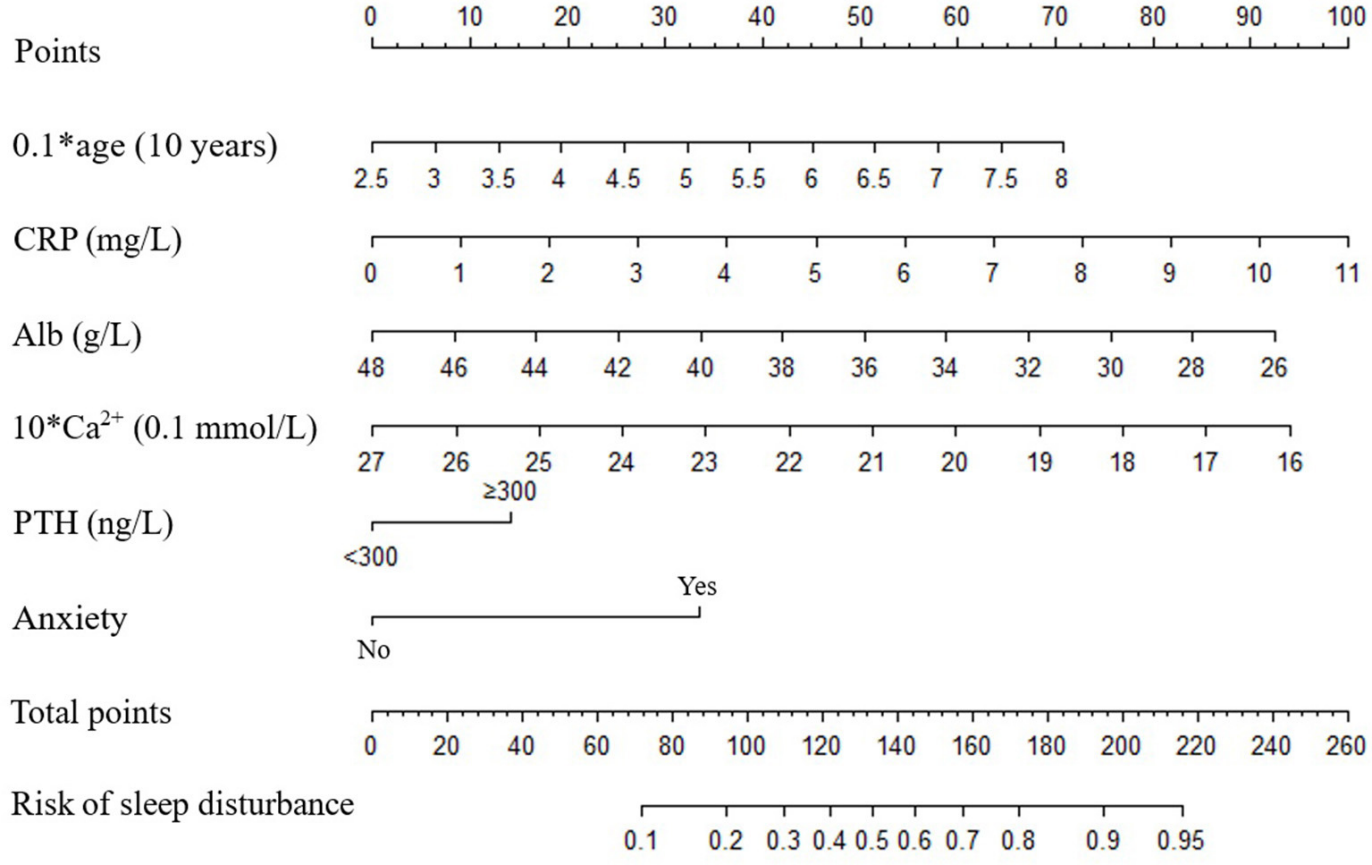
Nomograms predict the risk of sleep disturbance in patients with MHD. Alb, albumin; Ca^2+^, calcium; CRP, C-reactive protein; PTH, parathyroid hormone.

Age, duration of dialysis, CRP, TG, HDL-C, Alb, Ca^2+^, PTH, anxiety and depression were screened out to establish initial predictive model based on univariate binary logistic regression analyses between sleep disturbance or severe sleep disturbance and clinical characteristics, combined with variables considered to be meaningful in previous studies. Furthermore, age, CRP, Alb, Ca^2+^, PTH and anxiety were screened by two-way stepwise regression. A collinearity test showed that no collinearity existed among these variables. Multivariate binary logistic regression analysis was conducted with the selected age, CRP, Alb, Ca^2+^, PTH and anxiety as variables (Figure 1), and then the risk prediction model for sleep disturbance in MHD patients was developed, and a nomogram was constructed according to the parameters of the prediction model (Figure 2).

**Figure 3:**
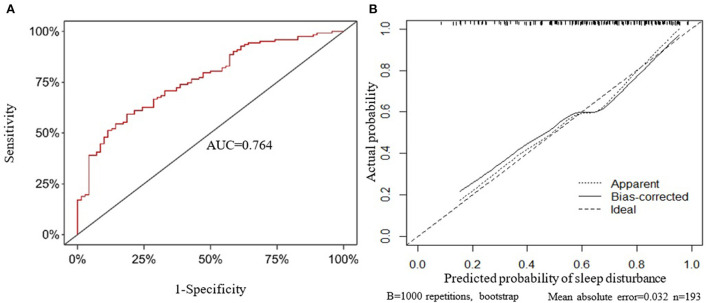
Internal validation of the prediction model. **(A)** The receiver operating characteristic curve. **(B)** Calibration curve. AUC, area under the curve.

Taking the original data of the prediction model as the data set, the ROC curve for predicting sleep disturbance in MHD patients was plotted, and the area under the curve was 0.764 (Figure 3A). The 1,000 bootstrap resampling procedure was used for internal validation of the prediction model, and the C-index after calibration was 0.736. Subsequently, calibration curve plots indicated that the calibration curve is approximately distributed along the reference line (y = x), with a mean absolute error of 0.032 (Figure 3B), the Hosmer-Lemeshow test (*P* = 0.434), demonstrated that the predictive probabilities of the model fit the actual probabilities well.

## Discussion

According to statistical analyses, CKD patients account for ~9.1% of the global population, and the prevalence of ESKD is as high as 550 people per million (23). At present, hemodialysis is the main treatment for ESKD patients except kidney transplantation. However, complications caused by hemodialysis, especially sleep disturbance, are often ignored (24). Studies have shown that there are many factors associated with sleep disturbance in MHD patients, such as gender, diet, depression and serum PTH levels (14, 16). In this study, we found that age, Alb and Ca^2+^ were independent risk factors for sleep disturbance in MHD patients, and confirmed that age, Alb and anxiety were associated with the severity of sleep disturbance in MHD patients. In this regard, we developed for the first time a risk prediction model for sleep disturbance in MHD patients.

The incidence of sleep disturbance in 193 MHD patients included in this study was 63.73%, which is similar to previous study results (25). Meanwhile, it should be noted that the incidence of severe sleep disturbance in MHD patients is as high as 26.42%. In addition, we found that prolonged sleep latency was the most common form of sleep disturbance in MHD patients, followed by daytime dysfunction with less use of sleep drugs. Previous study has also found that sleep latency has the greatest impact on MHD patients, and fewer patients use hypnotic drugs (16). This may be due to the increased daytime sleep in MHD patients during hemodialysis, resulting in difficulty in falling asleep at night, and thus significantly prolonged sleep latency. Additionally, MHD patients have a high prevalence of depression (26), and depression is related to prolonged sleep latency (27), which may be another reason for prolonged sleep latency in MHD patients. Meanwhile, studies have found that the severity of RLS is positively correlated with PSQI score in hemodialysis patients, and the sleep latency of hemodialysis patients with RLS is significantly prolonged (28, 29). Therefore, improving RLS may be an important direction to shorten the sleep latency of MHD patients. Furthermore, the renal function of MHD patients is almost lost, and it is widely believed that most drugs are mainly metabolized through kidney, which may make MHD patients resistant to drug administration. Therefore, it is known that sleep disturbance is common and serious in MHD patients, and shortening sleep latency may be an important strategy to improve sleep quality in MHD patients.

The association between poor sleep quality and old age has long been recognized among the general population and has also been shown in MHD patients (30, 31). In addition, a study has confirmed that sleep disturbance in MHD patients is independently related to age (32), and this result has also been verified in this study. In the current study, we found the age of MHD patients in the sleep disturbance group was significantly higher than those in the no sleep disturbance group, and further statistical analysis suggested that age was an independent risk factor for sleep disturbance in MHD patients. Furthermore, this study found that age was associated with the severity of sleep disturbance, showing that the risk of severe sleep disturbance increased by 0.476 times for each additional 10 years of age. Additionally, the risk of sleep disturbance was increased with the duration of dialysis (33). Although the present study found there was no significant difference in the duration of dialysis between the sleep disturbance and no sleep disturbance group, our results suggested that the duration of dialysis may be a risk factor for the severity of sleep disturbance in MHD patients. These results demonstrate that elderly patients with long dialysis age are more likely to have sleep disturbance.

Alb in MHD patients with sleep disturbance was significantly lower than that in without sleep disturbance, and the incidence of severe sleep disturbance was significantly reduced with the increase of Alb. Ling et al. (34) also found that hypo-albuminemia was associated with poor sleep quality in MHD patients. Furthermore, a cohort study found that Alb level was positively correlated with total sleep time in MHD patients, and that regulating hypo-albuminemia could improve their sleep disturbance (35). In addition, it is important to note that vitamin D deficiency is thought to be a potential cause of sleep disturbance (36). A meta-analysis showed that vitamin D deficiency is associated with a higher risk of sleep disturbance, and subgroup analysis suggested that vitamin D deficiency not only reduced the sleep quality, but also affected sleep duration (37). Importantly, the association between vitamin D and sleep disturbance was validated in MHD patients. Hejazian et al. (38) showed that vitamin D deficiency was an independent predictor of sleep disturbance in MHD patients. It is well known that Vitamin D is closely related to Ca^2+^ metabolism in the body. Meanwhile, the results of the current study showed a significant difference in Ca^2+^ between the sleep disturbance and the no sleep disturbance group. Therefore, regulating Ca^2+^ and Alb levels may be an important strategy for improving sleep quality in MHD patients.

The relationship between blood lipid and sleep quality in MHD patients is still unclear. A clinical study suggested that increased TG is associated with increased risk of sleep disturbance in MHD patients (39). In addition, a study has found that MHD patients with poor sleep quality have lower HDL-C levels than those with good sleep quality (25). However, a recent study suggested that TG and TC levels were not associated with the sleep disturbance in MHD patients (15). Although the results of the current study suggested that HDL-C may be a risk factor for severe sleep disturbance in MHD patients, there were no significant differences in TG, TC, HDL-C and LDL-C levels between the sleep disturbance and the no sleep disturbance group. These lines of evidence suggest that the role of lipid in sleep disturbance in MHD patients is ambiguous, and further studies will be needed to confirm the relationship.

Depression, anxiety and sleep disturbance are usually associated with a lower quality of life in MHD patients. Studies have found that depression is widespread in MHD patients and is a risk factor for sleep disturbance in MHD patients (26, 40). However, our study found that depression was not associated with sleep disturbance in MHD patients after adjusting for multiple covariates such as age, Alb and Ca^2+^. Such inconsistent results may be related to the fact that fewer MHD patients with depression were included in this study. It has been well recognized that sleep deprivation can lead to anxiety (41), which in turn can contribute to decreased sleep quality (42). In addition, Dikici et al. (43) found that anxiety was associated with sleep disturbance in MHD patients. Meanwhile, the results of this study also showed that anxiety can significantly increase the occurrence of severe sleep disturbance in MHD patients. Therefore, although our data did not find that anxiety and depression were independent risk factors for sleep disturbance in MHD patients, the impact of anxiety and depression on sleep disturbance requires our attention.

Although a series of previous studies have focused on prediction models related to sleep quality, no study has discussed the risk prediction models for sleep disturbance in MHD patients. Li et al. (44) incorporated Hb, respiratory rate, diastolic blood pressure, delirium and cardiovascular diseases into the prediction model for assessing the risk of sleep disturbance in ICU patients, which provided clinical decision support for improving the sleep quality of ICU patients. Yang et al. (45) developed a prediction model to assess the risk of postoperative sleep disturbance in non-cardiac surgery patients, and then to provide reasonable prevention and treatment measures. In addition, a study has developed and verified the mathematical model for predicting sleep latency and sleep duration, providing a basis for developing personalized optimal sleep plans (46). This study was the first to explore the risk prediction model of sleep disturbance in MHD patients, in order to improve their sleep quality.

In the present study, age, CRP, Alb, Ca^2+^, PTH and anxiety were used as predictors to develop a prediction model for sleep disturbance in MHD patients. As previously mentioned, age, Alb, Ca^2+^ and anxiety play important roles in MHD patients with sleep disturbance. The relationship between CRP and sleep quality has been demonstrated in multiple studies, with higher CRP levels indicating poorer sleep quality (47, 48). Furthermore, MHD patients are commonly associated with chronic inflammatory responses, and therefore often have elevated CRP levels. It is worth noting that CRP levels has been confirmed to be significantly negatively correlated with sleep quality of MHD patients (49). In addition, a clinical study has found that the incidence of sleep disturbance in MHD patients with hyperparathyroidism is significantly increased (50). Moreover, the sleep quality of MHD patients with hyperparathyroidism was significantly improved after parathyroidectomy (51). Therefore, although the current study did not find that CRP and PTH are the independent risk predictors for sleep disturbance in MHD patients, CRP and PTH were included in the prediction model based on previous evidence. Meanwhile, according to the actual clinical significance, age and Ca^2+^ were transformed into 0.1^*^ age and 10^*^Ca^2+^, respectively, and PTH was transformed into a categorical variable with a cut-off value of 300 ng/L. The internal validation of the prediction model was carried out by bootstrap resampling. The discrimination and calibration of the prediction model were evaluated with the C-index and calibration curve. In the present study, the prediction model has better discrimination and calibration. The C-index after calibration was 0.736, and the calibration curve was approximately distributed along the reference line (x = y).

There are some limitations in our study. First, this study had a single-center and cross-sectional design, which was not representative of the population and did not allow confirming causal relationship between sleep disturbance and risk factors. Further multicenter prospective cohort studies are needed to clarify the clinical value of risk factors in sleep disturbance in MHD patients. Second, our study developed a risk prediction model for sleep disturbance in MHD patients, providing a clinical guidance tool for improving sleep quality. However, this model has not been verified externally. In the future, external validation is needed to further improve the prediction efficiency of the model. Third, fewer patients with depression and anxiety were included in this study, which failed to adequately describe the relationship between depression, anxiety and sleep disturbance in MHD patients. Future studies should expand the sample size to further verify the interaction between depression, anxiety and sleep disturbance. Finally, our study relied on the scale to evaluate sleep quality, and we expect to use sleep monitoring devices such as polysomnography in the future, so as to provide better objective indicators for sleep monitoring of MHD patients.

In conclusion, sleep disturbance in MHD patients may be associated with age, Alb, Ca^2+^ and anxiety. In addition, we developed a risk prediction model for sleep disturbance in MHD patients, and early intervention with reversible predictors, such as CRP, Alb, Ca^2+^ and PTH, will contribute to the prevention and treatment of sleep disturbance in MHD patients. Subsequent studies should elucidate the exact pathogenesis of sleep disturbance in MHD patients and develop effective therapeutic strategies.

## Data availability statement

The raw data supporting the conclusions of this article will be made available by the authors, without undue reservation.

## Ethics statement

The studies involving human participants were reviewed and approved by the Ethics Committee of the Third Affiliated Hospital of Soochow University. The patients/participants provided their written informed consent to participate in this study.

## Author contributions

RX, LM, BZ, and RJ conceived the study. RX and LM performed the study and data analyses and drafted the manuscript. JN, YD, YS, and CY conducted the patient enrolment and collected the data. BZ and RJ revised the manuscript. All authors contributed to the article and approved the submitted version.

## Funding

This work was supported by the National Natural Science Foundation of China (81974171 and 81703482), Innovative and Entrepreneurial Team of Jiangsu Province (JSSCTD202144), the Program of Major Science and Technology Project of Changzhou Health Commission (No: ZD202101), and Changzhou Sci&Tech (Program Grant No. CJ20210090).

## Conflict of interest

The authors declare that the research was conducted in the absence of any commercial or financial relationships that could be construed as a potential conflict of interest.

## Publisher's note

All claims expressed in this article are solely those of the authors and do not necessarily represent those of their affiliated organizations, or those of the publisher, the editors and the reviewers. Any product that may be evaluated in this article, or claim that may be made by its manufacturer, is not guaranteed or endorsed by the publisher.

## References

[1] Jha V Garcia-Garcia G Iseki K Li Z Naicker S Plattner B. Chronic kidney disease: global dimension and perspectives. Lancet. (2013) 382:260–72. 10.1016/S0140-6736(13)60687-X 23727169

[2] Zhang L Wang F Wang L Wang W Liu B Liu J. Prevalence of chronic kidney disease in China: a cross-sectional survey. Lancet. (2012) 379:815–22. 10.1016/S0140-6736(12)60033-6 22386035

[3] Einollahi B Motalebi M Rostami Z Nemati E Salesi M. Sleep quality among Iranian hemodialysis patients: a multicenter study. Nephrourol Mon. (2015) 7:e23849. 10.5812/numonthly.23849 25738125 PMC4330687

[4] Chu G Choi P McDonald VM. Sleep disturbance and sleep-disordered breathing in hemodialysis patients. Semin Dial. (2018) 31:48–58. 10.1111/sdi.12617 28608937

[5] Al-Ali F Elshirbeny M Hamad A Kaddourah A Ghonimi T Ibrahim R. Prevalence of depression and sleep disorders in patients on dialysis: a cross-sectional study in Qatar. Int J Nephrol. (2021) 2021:5533416. 10.1155/2021/5533416 34136284 PMC8175178

[6] He S Zhu J Jiang W Ma J Li G He Y. Sleep disturbance, negative affect and health-related quality of life in patients with maintenance hemodialysis. Psychol Health Med. (2019) 24:294–304. 10.1080/13548506.2018.1515493 30160172

[7] Fitzpatrick J Kerns ES Kim ED Sozio SM Jaar BG Estrella MM. Functional outcomes of sleep predict cardiovascular intermediary outcomes and all-cause mortality in patients on incident hemodialysis. J Clin Sleep Med. (2021) 17:1707–15. 10.5664/jcsm.9304 33779539 PMC8656913

[8] Han Q Liu B Lin S Li J Liang P Fu S. Pittsburgh sleep quality index score predicts all-cause mortality in Chinese dialysis patients. Int Urol Nephrol. (2021) 53:2369–76. 10.1007/s11255-021-02842-6 33788131

[9] So JY Warburton KM Rosen IM. A guide to management of sleepiness in ESKD. Am J Kidney Dis. (2020) 75:782–92. 10.1053/j.ajkd.2019.09.010 31983503

[10] Brown RE Basheer R McKenna JT Strecker RE McCarley RW. Control of sleep and wakefulness. Physiol Rev. (2012) 92:1087–187. 10.1152/physrev.00032.2011 22811426 PMC3621793

[11] Bo Y Yeoh EK Guo C Zhang Z Tam T Chan TC. Sleep and the risk of chronic kidney disease: a cohort study. J Clin Sleep Med. (2019) 15:393–400. 10.5664/jcsm.7660 30853043 PMC6411180

[12] Yamamoto R Shinzawa M Isaka Y Yamakoshi E Imai E Ohashi Y. Sleep quality and sleep duration with CKD are associated with progression to ESKD. Clin J Am Soc Nephrol. (2018) 13:1825–32. 10.2215/CJN.01340118 30442866 PMC6302324

[13] Norozi Firoz M Shafipour V Jafari H Hosseini SH Yazdani-Charati J. Relationship of hemodialysis shift with sleep quality and depression in hemodialysis patients. Clin Nurs Res. (2019) 28:356–73. 10.1177/1054773817731852 28929785

[14] Yavuz D Demirag MD Yavuz R Karagoz Ozen DS Ramazanoglu ZB. 25-Hydroxy vitamin D level is associated with sleep disturbances in patients with chronic kidney disease on hemodialysis: a cross-sectional study. Turk J Med Sci. (2020) 50:298–303. 10.3906/sag-1908-87 31887852 PMC7164765

[15] Xu S Zou D Tang R Li S Chen W Wen L. Levels of trace blood elements associated with severe sleep disturbance in maintenance hemodialysis patients. Sleep Breath. (2021) 25:2007–13. 10.1007/s11325-021-02336-w 33666836

[16] Ho LL Chan YM Daud ZM. Dietary factors and sleep quality among hemodialysis patients in Malaysia. J Ren Nutr. (2022) 32:251–60. 10.1053/j.jrn.2021.02.003 33838975

[17] Hasannia E Derakhshanpour F Vakili MA. Effects of melatonin on salivary levels of cortisol and sleep quality of hemodialysis patients: a randomized clinical trial. Iran J Psychiatry. (2021) 16:305–11. 10.18502/ijps.v16i3.6256 34616464 PMC8452832

[18] Pan KC Hung SY Chen CI Lu CY Shih ML Huang CY. Social support as a mediator between sleep disturbances, depressive symptoms, and health-related quality of life in patients undergoing hemodialysis. PLoS ONE. (2019) 14:e0216045. 10.1371/journal.pone.0216045 31034497 PMC6488079

[19] Zigmond AS Snaith RP. The hospital anxiety and depression scale. Acta Psychiatr Scand. (1983) 67:361–70. 6880820 10.1111/j.1600-0447.1983.tb09716.x

[20] Deng Y He S Wang J. Validation of the hospital anxiety and depression scale and the perceived stress scale and psychological features in patients with periodontitis. J Periodontol. (2021) 92:1601–12. 10.1002/JPER.20-0756 33386608

[21] Buysse DJ Reynolds CF 3rd Monk TH Berman SR Kupfer DJ. The Pittsburgh sleep quality index: a new instrument for psychiatric practice and research. Psychiatry Res. (1989) 28:193–213. 2748771 10.1016/0165-1781(89)90047-4

[22] Yan DQ Huang YX Chen X Wang M Li J Luo D. Application of the Chinese version of the pittsburgh sleep quality index in people living with HIV: preliminary reliability and validity. Front Psychiatry. (2021) 12:676022. 10.3389/fpsyt.2021.676022 34295273 PMC8291081

[23] Collaboration GBDCKD. Global, regional, and national burden of chronic kidney disease, 1990–2017: a systematic analysis for the Global Burden of Disease Study 2017. Lancet. (2020) 395:709–33. 10.1016/S0140-6736(20)30045-3 32061315 PMC7049905

[24] Cheng E Evangelidis N Guha C Hanson CS Unruh M Wilkie M. Patient experiences of sleep in dialysis: systematic review of qualitative studies. Sleep Med. (2021) 80:66–76. 10.1016/j.sleep.2021.01.019 33571871

[25] Han B Zhu FX Shi C Wu HL Gu XH. Association between serum vitamin D levels and sleep disturbance in hemodialysis patients. Nutrients. (2017) 9:139. 10.3390/nu9020139 28216568 PMC5331570

[26] Othayq A Aqeeli A. Prevalence of depression and associated factors among hemodialyzed patients in Jazan area, Saudi Arabia: a cross-sectional study. Ment Illn. (2020) 12:1–5. 10.1108/MIJ-02-2020-0004 32742625 PMC7370954

[27] Lewis BA Gjerdingen D Schuver K Avery M Marcus BH. The effect of sleep pattern changes on postpartum depressive symptoms. BMC Womens Health. (2018) 18:12. 10.1186/s12905-017-0496-6 29316912 PMC5761144

[28] Orsal O Unsal A Balci-Alparslan G Duru P. Restless legs syndrome and sleep quality in patients on hemodialysis. Nephrol Nurs J. (2017) 44:167–76. 29165968

[29] Turk AC Ozkurt S Turgal E Sahin F. The association between the prevalence of restless leg syndrome, fatigue, and sleep quality in patients undergoing hemodialysis. Saudi Med J. (2018) 39:792–8. 10.15537/smj.2018.8.22398 30106417 PMC6194982

[30] Merikanto I Partonen T. Increase in eveningness and insufficient sleep among adults in population-based cross-sections from 2007 to 2017. Sleep Med. (2020) 75:368–79. 10.1016/j.sleep.2020.07.046 32950882

[31] Mirghaed MT Sepehrian R Rakhshan A Gorji H. Sleep quality in Iranian hemodialysis patients: a systematic review and meta-analysis. Iran J Nurs Midwifery Res. (2019) 24:403–9. 10.4103/ijnmr.IJNMR_184_18 31772913 PMC6875887

[32] Firoz MN Shafipour V Jafari H Hosseini SH Charati JY. Sleep quality and depression and their association with other factors in hemodialysis patients. Glob J Health Sci. (2016) 8:53485. 10.5539/gjhs.v8n8p121 27045404 PMC5016350

[33] Hamzi MA Hassani K Asseraji M El Kabbaj D. Insomnia in hemodialysis patients: a multicenter study from morocco. Saudi J Kidney Dis Transpl. (2017) 28:1112–8. 10.4103/1319-2442.215152 28937071

[34] Ling LL Chan YM Mat Daud Z. Serum potassium and handgrip strength as predictors of sleep quality among hemodialysis patients in Malaysia. Asia Pac J Clin Nutr. (2019) 28:401–10. 10.6133/apjcn.201906_28(2).0023 31192570

[35] Ezzat H Mohab A. Prevalence of sleep disorders among ESRD patients. Ren Fail. (2015) 37:1013–9. 10.3109/0886022X.2015.1044401 25959021

[36] Gominak SC Stumpf WE. The world epidemic of sleep disorders is linked to vitamin D deficiency. Med Hypotheses. (2012) 79:132–5. 10.1016/j.mehy.2012.03.031 22583560

[37] Gao Q Kou T Zhuang B Ren Y Dong X Wang Q. The association between vitamin D deficiency and sleep disorders: a systematic review and meta-analysis. Nutrients. (2018) 10:1395. 10.3390/nu10101395 30275418 PMC6213953

[38] Hejazian SM Ahmadian E Zununi Vahed S Faraji Gogani L Farnood F. The association of sleep quality and vitamin D levels in hemodialysis patients. Biomed Res Int. (2021) 2021:4612091. 10.1155/2021/4612091 34604382 PMC8481063

[39] Taraz M Khatami MR Hajiseyedjavadi M Farrokhian A Amini M Khalili H. Association between antiinflammatory cytokine, IL-10, and sleep quality in patients on maintenance hemodialysis. Hemodial Int. (2013) 17:382–90. 10.1111/hdi.12035 23490309

[40] Al Naamani Z Gormley K Noble H Santin O Al Maqbali M. Fatigue, anxiety, depression and sleep quality in patients undergoing haemodialysis. BMC Nephrol. (2021) 22:157. 10.1186/s12882-021-02349-3 33910523 PMC8080199

[41] Ben Simon E Rossi A Harvey AG Walker MP. Overanxious and underslept. Nat Hum Behav. (2020) 4:100–10. 10.1038/s41562-019-0754-8 31685950

[42] Gould CE Spira AP Liou-Johnson V Cassidy-Eagle E Kawai M Mashal N. Association of anxiety symptom clusters with sleep quality and daytime sleepiness. J Gerontol B Psychol Sci Soc Sci. (2018) 73:413–20. 10.1093/geronb/gbx020 28379498 PMC6074813

[43] Dikici S Bahadir A Baltaci D Ankarali H Eroglu M Ercan N. Association of anxiety, sleepiness, and sexual dysfunction with restless legs syndrome in hemodialysis patients. Hemodial Int. (2014) 18:809–18. 10.1111/hdi.12175 24865547

[44] Li Y Zhao L Yang C Yu Z Song J Zhou Q. Development and validation of a clinical prediction model for sleep disorders in the ICU: a retrospective cohort study. Front Neurosci. (2021) 15:644845. 10.3389/fnins.2021.644845 33935633 PMC8085546

[45] Yang S Zhang Q Xu Y Chen F Shen F Zhang Q. Development and validation of nomogram prediction model for postoperative sleep disturbance in patients undergoing non-cardiac surgery: a prospective cohort study. Nat Sci Sleep. (2021) 13:1473–83. 10.2147/NSS.S319339 34466046 PMC8403031

[46] Vital-Lopez FG Balkin TJ Reifman J. Models for predicting sleep latency and sleep duration. Sleep. (2021) 44:zsaa263. 10.1093/sleep/zsaa263 33249507

[47] Richardson MR Churilla JR. Sleep duration and C-reactive protein in US adults. South Med J. (2017) 110:314–7. 10.14423/SMJ.0000000000000632 28376532

[48] Huang WY Huang CC Chang CC Kor CT Chen TY Wu HM. Associations of self-reported sleep quality with circulating interferon gamma-inducible protein 10, interleukin 6, and high-sensitivity C-reactive protein in healthy menopausal women. PLoS ONE. (2017) 12:e0169216. 10.1371/journal.pone.0169216 28060925 PMC5218483

[49] Emami Zeydi A Jannati Y Darvishi Khezri H Gholipour Baradari A Espahbodi F Lesani M. Sleep quality and its correlation with serum C-reactive protein level in hemodialysis patients. Saudi J Kidney Dis Transpl. (2014) 25:750–5. 10.4103/1319-2442.134962 24969183

[50] De Santo RM Esposito MG Cesare CM Cice G Perna A Violetti E. High prevalence of sleep disorders in hemodialyzed patients requiring parathyroidectomy. J Ren Nutr. (2008) 18:52–5. 10.1053/j.jrn.2007.10.011 18089444

[51] Esposito MG Cesare CM De Santo RM Cice G Perna AF Violetti E. Parathyroidectomy improves the quality of sleep in maintenance hemodialysis patients with severe hyperparathyroidism. J Nephrol. (2008) 21:S92–6. 18446739

