# Supplementary material for: Risk factors and prediction model of sleep disturbance in patients with maintenance hemodialysis: A single center study
Source: Front Neurol. 2022 Jul 26;13:955352. doi: 10.3389/fneur.2022.955352 (PMC9360761; doi:10.3389/fneur.2022.955352)
Supplement: Supplementary file 1 [file Data_Sheet_1.docx]

Supplementary Material

# Supplementary Figures and Tables

##
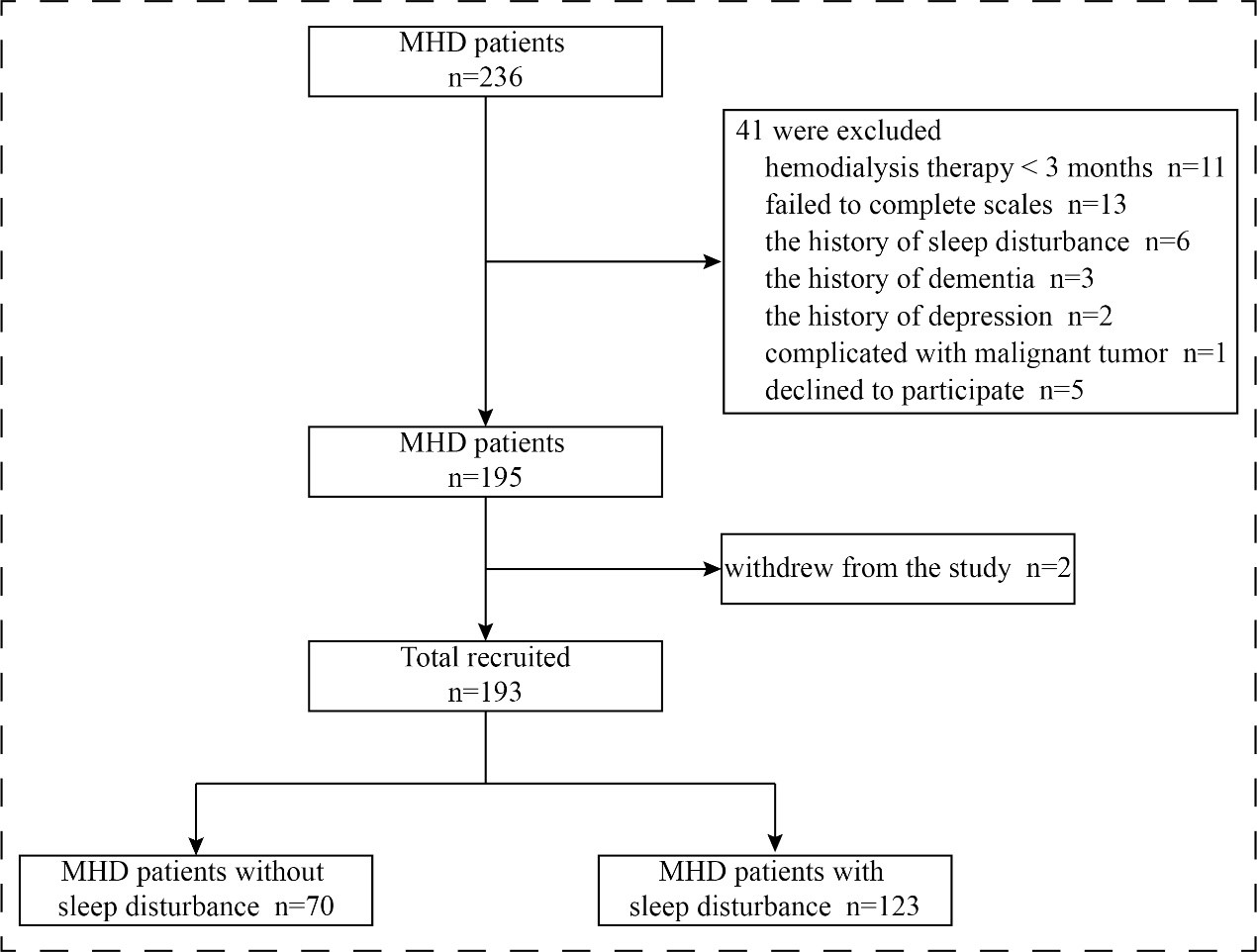
Supplementary Figures

**Supplementary Figure 1** Flow diagram to show recruitment of MHD patients. MHD, maintenance hemodialysis.


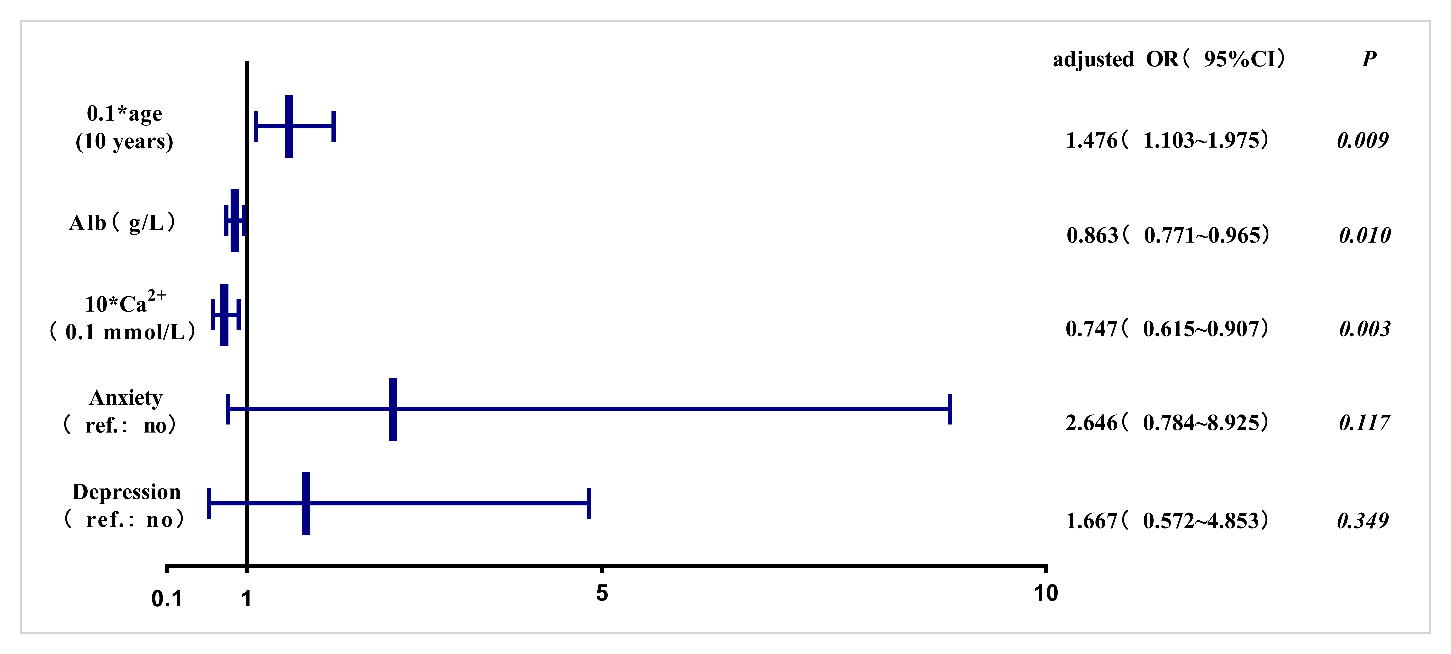
**Supplementary Figure 2** Multivariate binary logistic regression analysis between sleep disturbance of MHD patients and clinical characteristics. Alb, albumin; Ca^2+^, calcium; Ref., reference.

**Supplementary Figure 3** Multivariate binary logistic regression analysis between severe sleep disturbance of MHD patients and clinical characteristics. Alb, albumin; HDL-C, high-density lipoprotein cholesterol; ref., reference; TG, triglyceride.
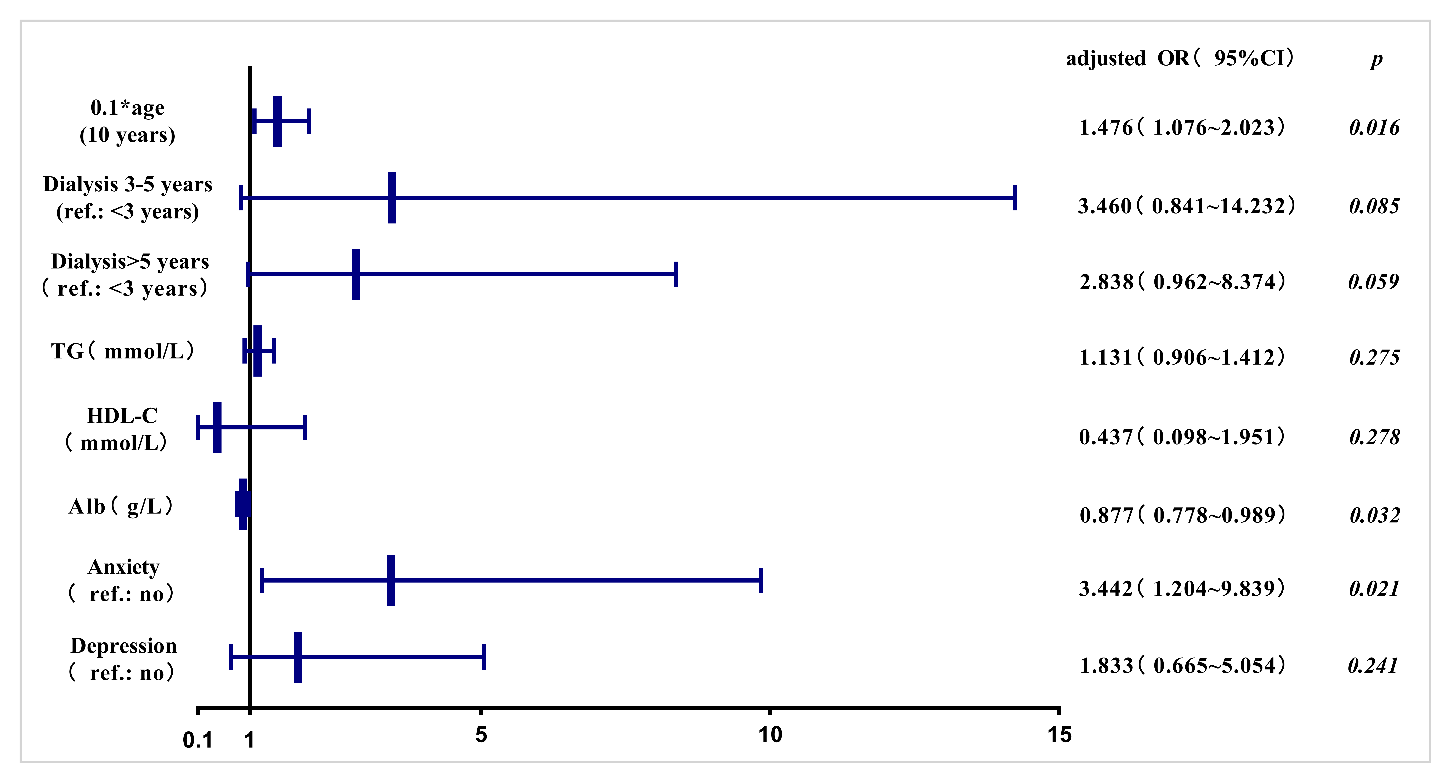


## Supplementary Tables

**Supplementary Table 1** Univariate binary logistic regression analyses between sleep disturbance of MHD patients and clinical characteristics.

| **Variables** | **β** | **SE** | **OR (95% *CI*)** | ***P* value** |
| --- | --- | --- | --- | --- |
| 0.1*age (10 years) | 0.361 | 0.136 | 1.435 (1.100-1.873) | 0.008 |
| Alb (g/L) | -0.190 | 0.055 | 0.827 (0.743-0.921) | 0.001 |
| 10*Ca^2+^ (0.1 mmol/L) | -0.314 | 0.095 | 0.731 (0.607-0.880) | 0.001 |
| Anxiety | 1.199 | 0.515 | 3.316 (1.208-9.106) | 0.020 |
| Depression | 0.929 | 0.454 | 2.531 (1.040-6.164) | 0.041 |

*Alb, albumin; Ca^2+^, calcium; MHD, maintenance hemodialysis.*

**Supplementary Table 2.** Clinical characteristics of MHD patients with or without severe sleep disturbance.

| **Variables** | **Total (n=193)** | **Severe sleep disturbance (n=51)** | **No severe sleep disturbance (n=142)** | **t/Z/χ^2^** | ***P* value** |
| --- | --- | --- | --- | --- | --- |
| Age (years) | 53.09±11.68 | 57.00±11.55 | 51.69±11.44 | 2.836 | 0.005^a^ |
| Male n (%) | 127 (65.8) | 31 (60.8) | 96 (67.6) | 0.776 | 0.378^c^ |
| BMI (kg/m^2^) | 21.93±3.26 | 21.85±3.06 | 21.97±3.34 | -0.218 | 0.828^a^ |
| Primary diseases |  |  |  | 1.471 | 0.689^c^ |
| Chronic glomerulo-nephritis n (%) | 40 (20.7) | 9 (17.6) | 31 (21.8) |  |  |
| Diabetic nephropathy n (%) | 26 (13.5) | 9 (17.6) | 17 (12.0) |  |  |
| Hypertensive nephropathy n (%) | 39 (20.2) | 9 (17.6) | 30 (21.1) |  |  |
| Others n (%) | 88 (45.6) | 24 (47.1) | 64 (45.1) |  |  |
| Duration of dialysis |  |  |  | -1.659 | 0.097^b^ |
| <3 years n (%) | 40 (20.7) | 5 (9.8) | 35 (24.6) |  |  |
| 3-5 years n (%) | 20 (10.4) | 7 (13.7) | 13 (9.2) |  |  |
| >5 years n (%) | 133 (68.9) | 39 (76.5) | 94 (66.2) |  |  |
| Smoking n (%) | 52 (26.9) | 14 (27.5) | 38 (26.8) | 0.009 | 0.924^c^ |
| Drinking n (%) | 19 (9.8) | 5 (9.8) | 14 (9.9) | 0.000 | 0.991^c^ |
| Married n (%) | 188 (97.4) | 50 (98.0) | 138 (97.2) | 0.000 | 1.000^c^ |
| High school or above n (%) | 103 (53.4) | 26 (51.0) | 77 (54.2) | 0.159 | 0.690^c^ |
| Hypertension n (%) | 163 (84.5) | 46 (90.2) | 117 (82.4) | 1.740 | 0.187^c^ |
| Diabetes mellitus n (%) | 35 (18.1) | 13 (25.5) | 22 (15.5) | 2.526 | 0.112^c^ |
| Cardiovascular and cerebrovascular diseases n (%) | 33 (17.1) | 10 (19.6) | 23 (16.2) | 0.308 | 0.579^c^ |
| Hb (g/L) | 108.73±18.47 | 110.12±18.79 | 108.23±18.39 | 0.627 | 0.532^a^ |
| WBC (10^9^/L) | 5.75 (4.85-6.92) | 6.04 (4.68-7.16) | 5.69 (4.85-6.86) | -0.910 | 0.363^b^ |
| RBC (10^12^/L) | 3.68±0.66 | 3.73±0.67 | 3.67±0.66 | 0.581 | 0.562^a^ |
| Hct (L/L) | 0.33±0.06 | 0.34±0.06 | 0.33±0.06 | 0.850 | 0.396^a^ |
| PLT (10^9^/L) | 178.00 (137.00-217.00) | 177 (118.00-224.00) | 178.00 (137.75-211.00) | -0.302 | 0.762^b^ |
| TG (mmol/L) | 1.60 (1.17-2.50) | 1.83 (1.30-2.90) | 1.56 (1.12-2.41) | -2.325 | 0.020^b^ |
| TC (mmol/L) | 4.11±0.97 | 4.01±0.91 | 4.14±0.99 | -0.878 | 0.381^a^ |
| HDL-C (mmol/L) | 0.95±0.27 | 0.88±0.26 | 0.98±0.27 | -2.408 | 0.017^a^ |
| LDL-C (mmol/L) | 2.30±0.74 | 2.19±0.66 | 2.34±0.77 | -1.201 | 0.231^a^ |
| Alb (g/L) | 38.20±3.16 | 37.17±2.92 | 38.57±3.17 | -2.756 | 0.006^a^ |
| P (mmol/L) | 1.97 (1.61-2.34) | 2.18 (1.57-2.46) | 1.96 (1.62-2.32) | -1.176 | 0.239^b^ |
| Ca^2+^ (mmol/L) | 2.28±0.18 | 2.27±0.16 | 2.28±0.19 | -0.205 | 0.838^a^ |
| CRP (mg/L) | 2.70 (2.20-3.40) | 2.90 (2.30-3.70) | 2.60 (2.10-3.30) | -1.486 | 0.137^b^ |
| PTH (ng/L) | 370.60 (197.35-609.25) | 353.70 (202.60-612.60) | 375.00 (193.10-607.98) | -0.355 | 0.723^b^ |
| Cr (umol/L) | 896.08±186.95 | 899.51±185.60 | 894.85±188.07 | 0.152 | 0.879^a^ |
| BUN (mmol/L) | 22.08 (18.11-26.30) | 23.41 (18.12-27.50) | 21.58 (18.09-26.01) | -1.134 | 0.257^b^ |
| Anxiety n (%) | 30 (15.5) | 16 (31.4) | 14 (9.9) | 13.229 | 0.000^c^ |
| Depression n (%) | 34 (17.6) | 15 (29.4) | 19 (13.4) | 6.645 | 0.010^c^ |

*Alb, albumin; BMI, body mass index; BUN, blood urea nitrogen; Ca^2+^, calcium; Cr, creatinine; CRP, C-reactive protein; Hb, hemoglobin; Hct, hematocrit; HDL-C, high-density lipoprotein cholesterol; LDL-C, low-density lipoprotein cholesterol; MHD, maintenance hemodialysis; P, phosphorus; PLT, blood platelet; PTH, parathyroid hormone; RBC, red blood cell; TC, total cholesterol; TG, triglyceride; WBC, white blood cell.*

*^a^ Calculated by Student's t test.*

*^b^ Calculated by Mann–Whitney U-test.*

*^c^ Calculated by chi-squared test.*

**Supplementary Table 3** Univariate binary logistic regression analyses between severe sleep disturbance of MHD patients and clinical characteristics.

| **Variables** | **β** | **SE** | **OR (95% *CI*)** | ***P* value** |
| --- | --- | --- | --- | --- |
| 0.1*age (10 years) | 0.401 | 0.146 | 1.493 (1.121-1.989) | 0.006 |
| Duration of dialysis |  |  |  |  |
| <3 years | ref. |  |  |  |
| 3-5 years | 1.327 | 0.670 | 3.769 (1.015-14.003) | 0.048 |
| >5 years | 1.066 | 0.515 | 2.904 (1.059-7.963) | 0.038 |
| TG (mmol/L) | 0.177 | 0.098 | 1.193 (0.984-1.447) | 0.072 |
| HDL-C (mmol/L) | -1.553 | 0.662 | 0.212 (0.058-0.774) | 0.019 |
| Alb (g/L) | -0.145 | 0.055 | 0.865 (0.778-0.963) | 0.008 |
| Anxiety | 1.430 | 0.413 | 4.180 (1.861-9.385) | 0.001 |
| Depression | 0.992 | 0.394 | 2.697 (1.246-5.838) | 0.012 |

*Alb, albumin; HDL-C, high-density lipoprotein cholesterol; MHD, maintenance hemodialysis; ref., reference; TG, triglyceride.*
